# Validation of the Modified Yale Food Addiction Scale 2.0 (mYFAS 2.0) in Spanish University Students

**DOI:** 10.3390/nu16203492

**Published:** 2024-10-15

**Authors:** Miguel López-Moreno, Carlos Marchena-Giráldez, Marta Garcés-Rimón, Marta Miguel-Castro, María Teresa Iglesias-López

**Affiliations:** 1 Instituto de Investigación en Ciencias de Alimentación, Consejo Superior de Investigaciones Científicas, Universidad Autónoma de Madrid, 28049 Madrid, Spain; miguel.lopez@ufv.es (M.L.-M.); marta.garces@ufv.es (M.G.-R.); marta.miguel@csic.es (M.M.-C.); 2 Grupo de Investigación en Biotecnología Alimentaria, Universidad Francisco de Vitoria, 28223 Madrid, Spain; 3 Faculty of Health Sciences, Universidad Francisco de Vitoria (UFV), Pozuelo de Alarcón, 28223 Madrid, Spain; 4 Faculty of Education and Psychology, Universidad Francisco de Vitoria (UFV), Pozuelo de Alarcón, 28223 Madrid, Spain; carlosalberto.marchena@ufv.es

**Keywords:** university students, food addiction, mYFAS 2.0, psychometric factor

## Abstract

Objective: The aim of this study was to explore the factorial structure, psychometric properties and association with anthropometric and clinical variables of the Modified Yale Addiction Scale 2.0 (mYFAS 2.0) in a Spanish university population. Methods: A cross-sectional study of n = 270 university students in Spain was conducted. Variables measured: mYFAS 2.0, Emotional Eater Questionnaire (EEQ), Alcohol Use Disorder Identification Test (AUDIT), Pittsburg Sleep Quality index (PSQI) and Perceived Stress Scale (PSS). In mYFAS 2.0 were performed an Exploratory Factor Analysis (EFA), an Unweight Least Square (ULS), a model fit using comparative fit index (CFI) and nomological validity. Results: EFA revealed that a two-factor structure is the most appropriate in a non-clinical population of Spanish university students. The factors accounted for 18.54% and 16.33% of the variance, and the correlation between them was moderate—0.487 (*p* < 0.001). This different structure from that reported in the original scale could be derived from the cultural characteristics and intrinsic eating habits of the study population. The correlation matrix reported an inverse association of mYFAS 2.0 with Body Mass Index (BMI). In addition, participants with a BMI below 25 showed a higher mYFAS 2.0 and EEQ score. Conclusions: These results suggest some changes in the psychometric assessment structure of the mYFAS 2.0 in a non-clinical population of university students, as well as the usefulness of this questionnaire to identify individuals with an adequate BMI but with compensatory behaviours that predispose to different eating disorders.

## 1. Introduction

Food addiction (FA) refers to the condition characterised by uncontrolled consumption of certain foods despite their negative consequences and the experience of loss of control over eating behaviour [1]. Numerous studies have described biological and behavioural similarities between FA and classic substances of abuse such as reward dysfunction, impulsivity and emotion dysregulation [2]. Not all foods are equally related to FA and in this sense foods rich in fats and refined carbohydrates are linked to a greater extent with this condition [3]. The combination of fat and refined carbohydrates appears to trigger a synergistic response in the neural activation of the reward system compared to the consumption of fats or refined carbohydrates separately [4]. This overactivation of reward mechanisms would be linked to overeating and thus to the risk of obesity, as first described in animals and later in humans [5,6].

In relation to the term FA, there is great controversy, since it is not formally considered an eating disorder by the Diagnostic and Statistics Manual of Mental Disorders-5 (DSM-5) [7]. Despite this, there is currently a validated questionnaire of interest for the diagnosis of FA, the Yale Food Addiction Scale (YFAS). The first version of the YFAS was published in 2009 based on the criteria for substance use disorders (SUD) included in the DSM-4 [7]. This 25-item version has demonstrated good consistency and incremental and convergent validity [8]. Subsequently, DSM-V involved the change of the SUD category to substance-related and addictive disorders (SRADs), the merging of the criteria for abuse and dependence, the inclusion of a new criterion linked to craving and the use of a continuum diagnosis of severity, resulting in YFAS 2.0 [9]. This new version with 35 items presents a unifactorial structure in which four new criteria are included: recurrent use in hazardous situations, failure in role obligation, use despite interpersonal/social consequences and craving. The modified version of the YFAS 2.0 (mYFAS-2) was developed and validated to simplify the previous scale by including 13 items for the diagnosis of FA [10].

The prevalence of FA determined by the YFAS is heterogeneous according to the study population. In this regard, a recent meta-analysis of 272 studies reported an overall FA prevalence of 20%, which differed according to the YFAS version used (21% with YFAS, 24% with YFAS 2.0 and 18% with mYFAS 2.0 [11] Likewise, these values were higher in the individuals with obesity (28%), clinically diagnosed anorexia (44%), clinically diagnosed bulimia (48%) and clinically diagnosed binge eating (55%) who were evaluated [12]. This situation confirms the findings of previous studies suggesting a J-shaped relationship between body mass index (BMI) and the prevalence of FA, since individuals with underweight and overweight/obesity, both conditions with negative implications on mental and physical health, show a higher prevalence of FA [13]. In a Spanish sample was assessed food addiction among several clinical populations with an eating disorder and gambling disorder and Yale Food Addiction Scale (YFAS) 2.0 was validated in a Spanish sample [14]. The university stage coincides with the transition from adolescence to young adulthood, which is a high-risk period for the development and persistence of eating disorders, body dissatisfaction and unhealthy weight control behaviours [15,16]. These eating disorders have deleterious effects on the university population, including poorer academic performance, comorbid mental disorders and an increased risk of mortality [17,18]. In relation to FA, previous studies have described how around 10% of the university population would have a diagnosis of FA using the YFAS [19]. This situation appears to be more prevalent among female students and in many cases co-occurs with other eating disorders [20]. Among university students, the occurrence of FA is associated with mental health disturbances, physical dissatisfaction, sleep disruption and a sedentary lifestyle [15,21]. Therefore, this situation highlights the need to develop a construct to further investigate this condition and to establish effective measures in the university population.

The aims of this study are (1) to explore the factor structure of the mYFAS 2.0 in healthy Spanish university population, (2) to determine its psychometric properties and (3) to assess the association between FA and anthropometric and clinical variables.

## 2. Methods

### 2.1. Study Design, Participants, and Recruitment

To validate the mYFAS scale, a minimum of 260 participants was required, following the guideline of 20 participants per scale item. Participants were recruited through social media by specifying the characteristics of the study, particularly the different domains of university lifestyle. Subsequently, interested participants enrolled through a form using Google Forms (Google LLC, Menlo Park, CA, USA). A total of 270 university health science students in Madrid (Spain) were recruited and agreed to participate in the study. Of these individuals, 93 (34.4%) were male with a mean age of 22.9 ± 3.1 years, while the mean age of the women (65.6%) was 21.4 ± 2.6 years. Following an explanation of the study and consent procedures, consenting students filled the questionnaire on paper and the respondents needed 30 min to fill in the questionnaire. Data collection was carried out between September–October 2021 and January 2022. The study fully complied with the Helsinki Declaration and was approved by the Research Ethics Committee of the University Francisco de Vitoria (19/2022); the date of approval was 22 March 2022.

The evaluation protocols were provided physically, and the data were uploaded onto a database for analysis using SPSS 26 software (SPSS Inc., Chicago, IL, USA). Consent was implied by agreeing to participate. Participants were recruited through social media by specifying the characteristics of the study; subsequently, interested participants enrolled through a form using Google Forms (Google LLC, Menlo Park, CA, USA). Prior to filling in the questionnaires, they had to accept to be included in the study and sign a written formal consent. The inclusion criteria were that they must be studying health sciences during the research period, with a mean age between 18 and 25 years. Students older than 25 years and students with health problems (non-communicable diseases or contagious diseases) were excluded from the study. Individuals were excluded if they did not properly finish the questionnaires.

Participants attended personally for anthropometric measurements. These were carried out using calibrated SECA^®^ digital scales (SECA Vogel & Halke, Hamburg, Germany) and SECA^®^ portable stadiometers (SECA Vogel & Halke, Hamburg, Germany). Participants were weighed barefoot and lightly clothed in kilograms to the nearest 100 g (0.1 kg), and height was measured with the subject fully erect, feet together, head in the Frankfort plane and arms hanging freely, to the nearest millimeter (0.1 cm). Body mass index (BMI) was calculated using the formula weight (kg)/[height (m)^2^]. Subjects were classified as underweight (BMI < 18.5 kg/m^2^), normal weight (BMI 18.5–24.9 kg/m^2^), overweight (BMI 25–29.9 kg/m^2^) or obese (BMI ≥ 30 kg/m^2^), according to World Health Organization (WHO) criteria [22].

### 2.2. Measures

#### 2.2.1. Modified Yale Food Addiction 2.0 (mYFAS 2.0)

The mYFAS 2.0 consists of 13 self-report items: 11 for food addiction symptoms and 2 for distress or impairment [10]. Specifically, the mYFAS assesses the experience of addictive eating behaviour in the past 12 months. This questionnaire contains an 8-point Likert scale, ranging from 0 (never) to 7 (every day). Each question has a different threshold: 0 = not meet, 1 = met. All mYFAS 2.0 questions are continuous and include two scoring options: a continuous symptom count reflecting the number of diagnostic criteria met by the participant and a diagnosis of FA based on the number of symptoms and clinically significant impairment or distress. The symptom count score (from 0 to 11) does not give clinical significance to the score whereas the diagnostic scoring option categorises individuals: No food addiction (1 or fewer symptoms), Mild food addiction (2 or 3 symptoms and clinical significance), Moderate food addiction (4 or 5 symptoms and clinical significance) and Severe food addiction (6 or more symptoms and clinical significance) [10].

#### 2.2.2. Emotional Eater Questionnaire (EEQ)

The questionnaire consists of 10 items on a 4-category Likert scale from 0 (never) to 3 (always). The original validation with a sample of people with obesity reported three subscales: lack of control in eating, high calorie food preference and feelings of guilt [23]. This questionnaire was validated in university students with a good internal consistency (α = 0.79) [24]. In our sample, the internal consistency of this measure was similar (α = 0.79). The subjects were classified into four groups: non-emotional eater (score 0–5), low emotional eater (score 6–10), emotional eater (score 11–20) and very emotional eater (score 21–30).

#### 2.2.3. Alcohol Use Disorder Identification Test (AUDIT)

The test was used to determine the alcohol consumption habits of the participants. The questionnaire was developed by the World Health Organisation to identify people with high-risk or harmful drinking habits by means of a simple screening test. The test consists of 10 items, 8 of which are on a Likert scale with 5 categories ranging from 0 (never/1 or 2 units) to 4 (daily/10 or more units). The remaining two items are also on a Likert scale but with 3 categories ordered from 0 to 2. The threshold established by García-Carretero et al. (2019) [25] was used to interpret the AUDIT score among Spanish university students: high-risk consumption (score 8–12 for males and score 6–12 for females) and probable alcohol dependence syndrome (ADS) (score ≧ 13 for both males and females). García Carretero et al. (2016) [26] reported the internal consistency of the AUDIT test in the Spanish population and in nursing students with α = 0.75 and 0.74, respectively [27]. The internal consistency of this measure found in the present study was α = 0.7.

#### 2.2.4. Pittsburg Sleep Quality Index (PSQI)

The PSQI is a 19-item questionnaire based on seven components of sleep (quality, onset latency, duration, efficiency, disturbance, use of sleep medication and daytime disturbance). Participants answer the various questions on typical nights during the previous month without distinguishing between weekday and weekend nights. Each component is scored from 0 to 3, with higher scores indicating worse sleep. The seven component scores are summed to produce a global score ranging from 0 to 21, with a score above 5 indicating poor sleep quality [28]. The internal consistency of this measure was found in the present study was α = 0.73.

#### 2.2.5. Perceived Stress Scale (PSS)

This scale assesses the level of perceived stress during the past month and consists of 14 items with a 5-point response scale (0 = never, to 4 = very often). The total score of the PSS is obtained by reversing the scores of items 4, 5, 6, 7, 9, 10 and 13 (in the following way: 0 = 4, 1 = 3, 2 = 2, 3 = 1, and 4 = 0) and then adding the scores of the 14 items. A higher score indicates a greater level of perceived stress [29,30]. The internal consistency of this measure for our study was α = 0.78.

### 2.3. Statistical Analysis

Descriptive analysis was carried out for the YFAS items. Distribution analysis and the Kolmogorov–Smirnov normality test were used to assess the distribution of variables. Secondly, Exploratory Factor Analysis (EFA) using Bartlett’s statistics and Kaiser–Meyer–Olkin (KMO) was calculated using a polychoric correlation matrix for the items in continuous format, and a point-biserial correlation matrix for the items in dichotomous format. Unweighted least squares (ULS) and oblique rotation were used as extraction methods. Parallel analyses based on minimum rank factor analysis and goodness of fit statistics were used to determine the number of factors. We used EFA to explore the internal structure of the questionnaire in a subclinical sample of young students of different ages. We did not know the background of the questionnaire structure in this population. Therefore, the best option is to use EFA and in the future we will analyse confirmatory factor analysis.

The model fit was assessed using the comparative fit index (CFI), root mean square error of approximation (RMSEA) with 95% of confidence interval, standardised root means square residual (SRMR) and Bayesian information criterion (BIC). Values above 0.95 for CFI and below 0.06 for RMSEA and SRMR are considered to indicate good model fit. For the BIC index, lower values indicate a better fit. Finally, the nomological validity was examined by calculating the correlation coefficients between mYFAS 2.0 and other related constructs, and *t*-test to calculate differences between all the measures according to the BMI. Correlation coefficients were interpreted using the following thresholds: trivial (r < 0.1), small (0.1 < r < 0.3), moderate (0.3 < r < 0.5), large (0.5 < r < 0.7), very large (0.7 < r < 0.9) and extremely perfect (0 ≥ 0.9). Cronbach’s alpha was used to analyse the internal consistency of the mYFAS 2.0. Factor software was employed to compute EFA and IBM SPSS^®^ version 22.0 for the remainder of the analysis [31].

## 3. Results

### 3.1. Descriptive Analysis

Regarding anthropometric parameters, the mean BMI was 22.9 ± 3.5 kg/m^2^ for women and 25.1 ± 3.5 kg/m^2^ for men. In addition, 33.7% of the participants were overweight/obese (BMI ≥ 25). Table 1 shows the main descriptive analysis of the items: means and SD for continuous format and percentage of participants meeting that criterion for dichotomic format. The average number of symptoms presented by the participants was 3.04 (SD = 2.42).

Univariate normality was exploring using the Kolmogorov–Smirnov test and was not assumed in any variable (*p* < 0.05). All the items reported positive skewness and leptokurtic distribution, which indicates homogenous and low scores in all the items.

### 3.2. Exploratory Factor Analysis

Bartlett’s statistics and KMO tests for the continuous items using polychromic matrix correlations show unacceptable values for the application of the factorial model (χ^2^ (55) = 1571.6; *p* < 0.001; KMO = 0.551). However, for the items in categorical format using point-biserial correlations matrix, the values were medium (χ^2^ (55) = 680.3; *p* < 0.001; KMO = 0.621). Thus, EFA was assessed using the dichotomic format.

Parallel analysis suggested two factors (α = 0.6) by using 500 random correlations and permutations of the raw data. Nevertheless, to find the most appropriate factorial structure, Table 2 shows the four models tested. The fit indices for the unifactorial model were unacceptable (α = 0.6). CFI index was higher as the model became more complex, but only > 0.95 for the four-factors model. RMSEA index was not less than 0.06 and RSMR index showed lower values as factors were added. However, the most parsimonious model, according to lower values of the BIC index, was the two-factors model, as recommended by parallel analysis.

As shown by the factor-loading for the two-factors models in Table 3, we represented the factor structure of the mYFAS 2.0. Factor 1 was composed of items 1, 2, 3 and 4, and accounted for 18.54% of the variance. Factor 2 was composed of items 7, 8, 9, 10, 11, 12 and 13. Nevertheless, the weight of items 4 and 8 in the respective factors was lower than 0.30. The factors accounted for 18.54% and 16.33% of the variance, and the correlation between them was moderate 0.487 (*p* < 0.001).

### 3.3. Nomological Validity and Internal Consistency

Table 4 and Table 5 shows descriptive statistics and the Spearman correlations computed between mYFAS 2.0, BMI, EEQ, PS, AUDIT and PQ to explore the association between related measures. mYFAS 2.0 was only related to BMI index in an inverse way, contrary to expectations. No other association was found between the mYFAS 2.0 and the rest of the measures. Internal consistency of the mYFAS 2.0 (α = 0.704) was acceptable.

As shown in Table 6, the results from the *t* test showed significant differences between participants with BMI < 25 and participants with BMI 25 regarding food addiction, emotional eating scores and in the scores of the sleep questionnaire. In all the cases, participants with BMI < 25 showed higher scores [11]. The effect size was R_XY_^2^ = 0.03 for food addiction and emotional eating and R_XY_^2^ = 0.02 for the sleep questionnaire.

## 4. Discussion

The aims of this study were to explore the factor structure and psychometric properties of the mYFAS 2.0 in non-clinical Spanish university students. mYFAS 2.0 was initially validated on subjects with an average BMI of 26.67 (overweight) [10]. Subsequently, this questionnaire has been validated on Taiwan and Chinese university students with good internal consistency, reliability and construct validity [32,33]. Therefore, it is necessary to examine the factor structure, reliability and validity of the mYFAS 2.0 in a non-clinical Spanish population, specifically in university students.

In the present study, the mYFAS 2.0 scale in Spanish university students showed a two-factor structural solution with adequate psychometric characteristics. This structure is different from that reported in the original scale, as a good fit was confirmed for a one-factor model [10]. In non-clinical population, the Italian, French and Czech version of the mYFAS 2.0 showed that the one-factor structure was also the best solution after the CFI with good psychometric properties [34,35,36]. So far, few studies have been carried out in a university population. Similar to our work, the Chinese mYFAS 2.0 scale showed a two-factor structure after confirmatory factor analysis (CFA) [33]. In contrast, in Taiwanese students was observed a better construct validity for a single-factor structure of the YFAS [32]. This discordance could be derived from the cultural characteristics and intrinsic dietary habits of each of the study populations. The DSM-5 includes 11 diagnostic criteria for substance use disorder that can be classified into four categories: impaired control, social problems, risky use and physical dependence [7]. The social problems and physical dependence categories include the first four items corresponding to factor 1 of this study (Items 1, 2, 3 and 4), which represent the psychosocial consequences of emotional eating. On the other hand, the 7 items in factor 2 (Items 7, 8, 9, 10, 11, 12 and 13) are related to the behavioural dimension of food. Therefore, according to the analysis of the items and the implications of the factors, the mYFAS 2.0 in the study population was divided into two factors, based on the emotional implications of the diet and on its behavioural aspect.

The mYFAS score was inversely related to BMI. These results could be due to the characteristics of the study population. We must take into consideration that the occurrence of food addiction is higher among underweight and overweight subjects, compared to subjects with normal weight [13]. In this sense, psychological distress is a relevant risk factor linked to food addiction, and this association seems to be mediated by self-control [37]. Likewise, previous studies in college students have described the relation between food addiction and weight dissatisfaction, which in the short term could induce a reduction of energy intake as a compensatory behaviour [15,38]. Among college students, perfectionism appears to be a proxy for emotional eating and binge eating, which similarly to the present work is linked to lower body weight [39]. Therefore, the results obtained could reflect the concurrence of behaviours targeted at reducing energy intake in university students with a higher mYFAS score, which triggers a decrease in BMI. This situation could be the prelude to conditions linked to a severe restriction of dietary intake, such as eating disorders [14]. Therefore, the mYFAS 2.0 could be a very interesting screening tool to identify normal-weight individuals at increased risk of eating disorders.

## 5. Limitations

The study has some limitations. First, the sample size of the study was limited and predominantly women. Additionally, KMO values report sampling biases that will be corrected in future factorial confirmatory studies. Second, the low internal consistency observed could be linked to the lack of correlation between mYFAS 2.0 and emotional eating. However, this phenomenon has been reported in previous studies, since women represent a higher proportion in university degrees in health sciences (López-Moreno et al., 2021) [14,40]. Third, we did not observe a normal distribution of the data and yielded low variability between all of them. Fourth, mYFAS 2.0 models may differ between university students and the general population, requiring analysis in different Spanish non-clinical samples. Fourth, the study utilized a voluntary sampling technique, which may introduce selection bias and limit the generalizability of the findings to the broader population. Finally, there is currently a lack of consensus on food addiction and the physiological mechanisms involved, which makes it difficult to interpret the results obtained.

## 6. Conclusions

These results suggest some changes in the psychometric assessment structure of the mYFAS 2.0 in a non-clinical population of Spanish university students. In particular, the two-factor structure showed adequate goodness of fit, consistency and validity based on its relationship with other related variables in this population. It also suggests the usefulness of the questionnaire to identify individuals with an adequate BMI but with compensatory behaviours predisposing to different eating disorders.

Additional research is needed to understand university population behaviour with respect to food addiction by using mYFAS 2.0 and any potential health effects.

## Figures and Tables

**Table 1:** Descriptive statistics of mYFAS 2.0 items.

Item	*M*	*SD*	Percentage	Skewness	Kurtosis
1	0.49	0.874	30.7	2.227	6.905
2	1.02	1.426	49.3	1.897	4.089
3	0.3	0.902	15.2	4.368	24.139
4	0.6	1.263	25.2	2.448	5.737
5	0.5	1.175	23.3	3.351	13.487
6	0.37	1.102	17.4	4.338	21.101
7	0.18	0.715	9.6	6.245	48.298
8	0.63	1.32	31.1	3.094	10.901
9	0.73	1.488	31.5	2.67	7.147
10	0.67	1.087	36.3	1.9	3.772
11	1.19	1.875	42.6	1.728	2.125
12	0.32	0.889	17.4	3.806	16.329
13	0.27	0.808	15.2	3.887	16.801

*M*: mean value. *SD*: standart deviation.

**Table 2:** Fit index values for mYFAS 2.0 models.

Model	χ^2^	df	CFI	RMSEA (95% CI)	RMSR	BIC
1 factor	255.486	44	0.661	0.134 (0.1033–0.1476)	0.1414	378.651
2 factors	117.724	34	0.866	0.096 (0.0644–0.1146)	0.0759	302.472
3 factors	72.583	25	0.924	0.084 (0.0583–0.1046)	0.0493	318.914
4 factors	34.904	17	0.971	0.063 (0.0274–0.0807)	0.0301	342.817

**Table 3:** Factor structure of the mYFAS 2.0.

Items	F1	F2
1. I ate to the point where I felt physically ill	0.838	−0.111
2. I spent more time feeling sluggish or tired from overeating	−0.388	0.125
3. I avoided work. school or social activities because I was afraid, I would overeat there	0.964	0.049
4. If I had emotional problems because I had not eaten certain foods, I would eat those foods to feel better	−0.212	0.034
7. My overeating got in the way of me taking care of my family or doing household chores	0.268	0.339
8. I kept eating in the same way even though my eating caused emotional problems	0.218	0.252
9. Eating the same amount of food did not give me as much enjoyment as it used to	−0.010	0.520
10. I had such strong urges to eat certain foods that I could not think of anything else	−0.038	−0.403
11. I tried and failed to cut down on or stop eating certain foods	−0.059	0.627
12. I was so distracted by eating that I could have been hurt (e.g., when driving a car, crossing the street and operating machinery)	−0.083	−0.433
13. My friends or family were worried about how much I overate	0.351	0.401

**Table 4:** Descriptive statistics of all measures.

Measure	*M*	*SD*	Range
YFAS	6.4	6.14	[0–27]
BMI	23.7	3.61	[17–39]
EEQ	8.6	4.79	[0–25]
PS	22.5	7.87	[3–40]
AUDIT	5.7	4.93	[0–26]
PQ	8.7	3.92	[1–19]

*M*: mean value; *SD*: standard deviation; YFAS, Food Addiction Scale; BMI, Body Mass Index; EEQ, Emotional Eating Questionnaire; PS, Perceived Stress Questionnaire; AUDIT, Alcohol Consumption; PQ, Pittsburgh Sleep Questionnaire.

**Table 5:** Spearman correlation among variables.

	1	2	3	4	5	6
1. YFAS	-	−0.220 **	0.037	−0.022	0.046	−0.062
2. BMI		-	−0.142 *	−0.046	0.027	−0.121*
3. EEQ			-	−0.009	−0.054	0.067
4. PS				-	−0.126 *	0.052
5. AUDIT					-	−0.087
6. PQ						-

* *p* < 0.05; ** *p* < 0.001. YFAS, Food Addiction Scale; BMI, Body Mass Index; EEQ, Emotional Eating Questionnaire; PS, Perceived Stress Questionnaire; AUDIT, Alcohol Consumption; PQ, Pittsburgh Sleep Questionnaire.

**Table 6:** Differences in all measures according to BMI.

	BMI < 25 (N = 179)		BMI > 25 (N = 91)		*t* Test
	*M*	*SD*	*M*	*SD*	
YFAS	7.23	6.34	4.75	5.37	3.36 **
EEQ	9.25	5.19	7.30	3.58	3.638 **
PS	22.50	7.76	22.35	8.12	0.149
AUDIT	5.47	4.86	6.15	5.05	−1.07
PQ	9.19	3.97	7.87	3.70	2.644 **

** *p* < 0.001. YFAS, Food Addiction Scale; BMI, Body Mass Index; EEQ, Emotional Eating Questionnaire; PS, Perceived Stress Questionnaire; AUDIT, Alcohol Consumption; PQ, Pittsburgh Sleep Questionnaire; *M*: mean values; *SD*: standard deviation.

## Data Availability

The original contributions presented in the study are included in the article, further inquiries can be directed to the corresponding author.

## References

[1] Oliveira J., Colombarolli M.S., Cordás T.A. (2021). Prevalence and correlates of food addiction: Systematic review of studies with the YFAS 2.0. Obes. Res. Clin. Pract..

[2] Vasiliu O. (2021). Current Status of Evidence for a New Diagnosis: Food Addiction-A Literature Review. Front. Psychiatry.

[3] Gordon E., Ariel-Donges A., Bauman V., Merlo L. (2018). What Is the Evidence for “Food Addiction?” A Systematic Review. Nutrients.

[4] DiFeliceantonio A.G., Coppin G., Rigoux L., Edwin Thanarajah S., Dagher A., Tittgemeyer M., Small D.M. (2018). Supra-Additive Effects of Combining Fat and Carbohydrate on Food Reward. Cell Metab..

[5] Sclafani A., Springer D. (1976). Dietary obesity in adult rats: Similarities to hypothalamic and human obesity syndromes. Physiol. Behav..

[6] Hall K.D., Ayuketah A., Brychta R., Cai H., Cassimatis T., Chen K.Y., Chung S.T., Costa E., Courville A., Darcey V. (2019). Ultra-Processed Diets Cause Excess Calorie Intake and Weight Gain: An Inpatient Randomized Controlled Trial of Ad Libitum Food Intake. Cell Metab..

[7] American Phychiatric Association (2013). Diagnostic and Statistical Manual of Mental Disorders.

[8] Gearhardt A.N., Corbin W.R., Brownell K.D. (2009). Preliminary validation of the Yale Food Addiction Scale. Appetite.

[9] Gearhardt A.N., Corbin W.R., Brownell K.D. (2016). Development of the Yale Food Addiction Scale Version 2.0. Psychol. Addict. Behav. J. Soc. Psychol. Addict. Behav..

[10] Schulte E.M., Gearhardt A.N. (2017). Development of the Modified Yale Food Addiction Scale Version 2.0. Eur. Eat. Disord. Rev..

[11] Diotaiuti P., Valente G., Mancone S. (2021). Development and Preliminary Italian Validation of the Emergency Response and Psychological Adjustment Scale. Front. Psicol..

[12] Praxedes D.R.S., Silva-Júnior A.E., Macena M.L., Oliveira A.D., Cardoso K.S., Nunes L.O., Monteiro M.B., Melo I.S.V., Gearhardt A.N., Bueno N.B. (2022). Prevalence of food addiction determined by the Yale Food Addiction Scale and associated factors: A systematic review with meta-analysis. Eur. Eat. Disord. Rev..

[13] Hauck C., Weiß A., Schulte E.M., Meule A., Ellrott T. (2017). Prevalence of “Food Addiction” as Measured with the Yale Food Addiction Scale 2.0 in a Representative German Sample and Its Association with Sex, Age and Weight Categories. Obes. Facts.

[14] Granero R., Jiménez-Murcia S., Gerhardt A.N., Agüera Z., Aymamí N., Gómez-Peña M., Lozano-Madrid M., Mallorquí-Bagué N., Mestre-Bach G., Neto-Antao M.I. (2018). Validation of the Spanish version of the Yale Food Addiction Scale 2.0 (YFAS 2.0) and clinical correlates in a sample of eating disorder, gambling disorder, and healthy control participants. Front. Psychiatry.

[15] Wu Y.-K., Zimmer C., Munn-Chernoff M.A., Baker J.H. (2020). Association between food addiction and body dissatisfaction among college students: The mediating role of eating expectancies. Eat. Behav..

[16] Grammer A.C., Fitzsimmons-Craft E.E., Laing O., De Pietro B., Wilfley D.E. (2020). Eating Disorders on College Campuses in the United States: Current Insight on Screening, Prevention, and Treatment. Curr. Psychopharmacol..

[17] Claydon E., Zullig K.J. (2020). Eating disorders and academic performance among college students. J. Am. Coll. Health J. ACH.

[18] Arcelus J., Mitchell A.J., Wales J., Nielsen S. (2011). Mortality rates in patients with anorexia nervosa and other eating disorders. A meta-analysis of 36 studies. Arch. Gen. Psychiatry.

[19] Yu Z., Tan M. (2016). Disordered Eating Behaviors and Food Addiction among Nutrition Major College Students. Nutrients.

[20] Yu Z., Indelicato N.A., Fuglestad P., Tan M., Bane L., Stice C. (2018). Sex differences in disordered eating and food addiction among college students. Appetite.

[21] Romero-Blanco C., Hernández-Martínez A., Parra-Fernández M.L., Onieva-Zafra M.D., Prado-Laguna M.D.C., Rodríguez-Almagro J. (2021). Food Addiction and Lifestyle Habits among University Students. Nutrients.

[22] WHO (2000). Obesity: Preventing and Managing the Global Epidemic.

[23] Garaulet M., Canteras M., Morales E., López-Guimera G., Sánchez-Carracedo D., Corbalán-Tutau M.D. (2012). Validation of a questionnaire on emotional eating for use in cases of obesity: The Emotional Eater Questionnaire (EEQ). Nutr. Hosp..

[24] Bernabéu E., Marchena C., Iglesias M.T. (2020). Factor Structure and Psychometric Properties of Emotional Eater Questionnaire (EEQ) in Spanish Colleges. Int. J. Environ. Res. Public Health.

[25] García-Carretero M.Á., Moreno-Hierro L., Martínez M.R., de los Ángeles Jordán-Quintero M., Morales-García N., O’Ferrall-González C. (2019). Alcohol consumption patterns of university students of health sciences. Enferm. Clin..

[26] Carretero M.Á., Ruiz J.P., Delgado J.M., González C.O. (2016). Validación del test para la identificación de trastornos por uso de alcohol en población universitaria: AUDIT y AUDIT-C. Adicciones.

[27] Carretero M.Á.G., Ruiz J.P.N., Delgado J.M.M., González C.O. (2016). Validation of the alcohol use disorders identification test in university students: AUDIT and AUDIT-C. Adicciones.

[28] Buysse D.J., Reynolds C.F., Monk T.H., Berman S.R., Kupfer D.J. (1989). The Pittsburgh Sleep Quality Index: A new instrument for psychiatric practice and research. Psychiatry Res..

[29] Cohen S., Kamarck T., Mermelstein R. (1983). A global measure of perceived stress. J. Health Soc. Behav..

[30] Remor E. (2006). Psychometric properties of a European Spanish version of the Perceived Stress Scale (PSS). Span. J. Psychol..

[31] Ferrando P.J., Lorenzo-Seva U. (2014). El análisis factorial exploratorio de los ítems: Algunas consideraciones adicionales. An. Psicol..

[32] Chen I.-H., Huang P.-C., Lin Y.-C., Gan W.Y., Fan C.-W., Yang W.-C., Tung S.E.H., Poon W.C., Griffiths M.D., Lin C.-Y. (2022). The Yale Food Addiction Scale 2.0 and the modified Yale Food Addiction Scale 2.0 in Taiwan: Factor structure and concurrent validity. Front. Psychiatry.

[33] Zhang H., Tong T., Gao Y., Liang C., Yu H., Li S., Yan X., Wang L. (2021). Translation of the Chinese version of the modified Yale Food Addiction Scale 2.0 and its validation among college students. J. Eat. Disord..

[34] Imperatori C., Fabbricatore M., Lester D., Manzoni G.M., Castelnuovo G., Raimondi G., Innamorati M. (2019). Psychometric properties of the modified Yale Food Addiction Scale Version 2.0 in an Italian non-clinical sample. Eat. Weight Disord. EWD.

[35] Pipová H., Kaščáková N., Fürstová J., Tavel P. (2020). Development of the Modified Yale Food Addiction Scale Version 2.0 summary version in a representative sample of Czech population. J. Eat. Disord..

[36] Brunault P., Berthoz S., Gearhardt A.N., Gierski F., Kaladjian A., Bertin E., Tchernof A., Biertho L., de Luca A., Hankard R. (2020). The Modified Yale Food Addiction Scale 2.0: Validation Among Non-Clinical and Clinical French-Speaking Samples and Comparison With the Full Yale Food Addiction Scale 2.0. Front. Psychiatry.

[37] Luo Y., Zhang Y., Sun X., Dong J., Wu J., Lin X. (2022). Mediating effect of self-control in the relationship between psychological distress and food addiction among college students. Appetite.

[38] Gonçalves S., Félix S., Martins F., Lapenta O., Machado B.C., Conceição E.M. (2022). Food Addiction Problems in College Students: The Relationship between Weight-Related Variables, Eating Habits, and Food Choices. Int. J. Environ. Res. Public Health.

[39] Bernabéu-Brotóns E., Marchena-Giráldez C. (2022). Emotional Eating and Perfectionism as Predictors of Symptoms of Binge Eating Disorder: The Role of Perfectionism as a Mediator between Emotional Eating and Body Mass Index. Nutrients.

[40] López-Moreno M., Garcés-Rimón M., Miguel M., Iglesias-López M. (2021). Influence of eating habits and alcohol consumption on the academic performance among a university population in the community of Madrid: A pilot study. Heliyon.

